# Spike-timing computation properties of a feed-forward neural network model

**DOI:** 10.3389/fncom.2014.00005

**Published:** 2014-01-28

**Authors:** Drew B. Sinha, Noah M. Ledbetter, Dennis L. Barbour

**Affiliations:** Laboratory of Sensory Neuroscience and Neuroengineering, Department of Biomedical Engineering, Washington University in St. LouisSt. Louis, MO, USA

**Keywords:** spike-timing dependent plasticity (STDP), computational modeling, network connectivity, biological neural networks, microcircuits

## Abstract

Brain function is characterized by dynamical interactions among networks of neurons. These interactions are mediated by network topology at many scales ranging from microcircuits to brain areas. Understanding how networks operate can be aided by understanding how the transformation of inputs depends upon network connectivity patterns, e.g., serial and parallel pathways. To tractably determine how single synapses or groups of synapses in such pathways shape these transformations, we modeled feed-forward networks of 7–22 neurons in which synaptic strength changed according to a spike-timing dependent plasticity (STDP) rule. We investigated how activity varied when dynamics were perturbed by an activity-dependent electrical stimulation protocol (spike-triggered stimulation; STS) in networks of different topologies and background input correlations. STS can successfully reorganize functional brain networks *in vivo*, but with a variability in effectiveness that may derive partially from the underlying network topology. In a simulated network with a single disynaptic pathway driven by uncorrelated background activity, structured spike-timing relationships between polysynaptically connected neurons were not observed. When background activity was correlated or parallel disynaptic pathways were added, however, robust polysynaptic spike timing relationships were observed, and application of STS yielded predictable changes in synaptic strengths and spike-timing relationships. These observations suggest that precise input-related or topologically induced temporal relationships in network activity are necessary for polysynaptic signal propagation. Such constraints for polysynaptic computation suggest potential roles for higher-order topological structure in network organization, such as maintaining polysynaptic correlation in the face of relatively weak synapses.

## Introduction

The properties of a neural network, including its connection structure and the efficacy of transmission between neurons, shape and constrain its computational properties. These properties are also likely to be a major determinant for revealing the computational role of specific neural circuits (Fiete et al., 2010). Unfortunately, neural circuit structure is extremely challenging to discern *in vivo* with any precision. Functional manifestations of network activity, however, are much easier to evaluate *in vivo* by measuring network or organism behavior. Conveniently, the functioning of neural networks can be perturbed and measured using external manipulation.

Techniques that use external stimulation to persistently modify neural circuitry rely upon neuroplasticity mechanisms. Neuroplasticity, the ability for the synaptic efficacies to change over time, has long been thought to drive long-term changes in neural systems and to form the basis of learning by allowing network-wide spiking activity to modulate network function via tuning individual synaptic efficacies (Hebb, 1949; Bliss and Collingridge, 1993). In spike-timing dependent plasticity (STDP), Hebbian-like plasticity is induced by precise spike timing between pre- and post-synaptic neurons (Abbott and Nelson, 2000; Dan and Poo, 2004; Morrison et al., 2008). STDP has classically been induced *in vitro* by isolating monosynaptically connected neurons in brain slices and stimulating pre- and post-synaptic neurons sequentially at various time delays (Markram et al., 1997; Bi and Poo, 1998; Froemke and Dan, 2002). STDP, however, also been implicated in changes induced in neural pathways during *in vivo* stimulation (Caporale and Dan, 2008; Froemke et al., 2013).

One promising class of *in vivo* stimulation techniques for manipulating neural function is spike-triggered stimulation (STS). The goal of STS is to activate natural STDP by artificially synchronizing the activity of two sites in order to persistently increase the connectivity between them (Jackson et al., 2006; Rebesco et al., 2010; Guggenmos et al., 2013; Song et al., 2013). Unfortunately, the effects of STS are quite variable when applied to *in vivo* networks, implying that particular network structures may be an important contributor to the STS effects that have been observed. Understanding the neural network configurations that can give rise to successful STS manipulation is an important step toward developing a novel methodology capable of discerning aspects of neural network connection structure *in vivo*. Furthermore, understanding which neural network structures are particularly amenable to external manipulation will reveal the most productive strategies for therapeutically rewiring brain circuits following injury.

While experimental studies have established that direct neural network manipulation is possible *in vivo*, few attempts have been made to develop frameworks that can predict changes in network activity due to experimenter-induced external perturbation. For example, network topology appears to influence both ongoing and persistent network controllability (Whalen et al., 2013). Systematic analyses have likely not been pursued in the experimental setting because, for networks of more than a handful of live neurons, it is infeasible to elucidate extensive network properties with current methodologies. In neurophysiological studies the analysis of network changes emphasizes alterations in quantities usually formulated in terms of single-unit and two-unit responses, such as interspike interval distributions, Fano factor analysis or notions of correlation between two neurons (Ahissar et al., 1992; Honey and Sporns, 2008; Rebesco et al., 2010; Song et al., 2013). Only recently have complex multi-unit correlation or information theoretic metrics been extensively developed and applied to neural systems (Ohiorhenuan and Victor, 2011; Song et al., 2013). Moreover, even in relatively small networks of tens or hundreds of neurons, the combinatorial explosion of pair-wise (or more generally, *n*-wise) quantities makes the selection of relevant features useful for understanding individual intranetwork interactions challenging. In addition, even if such quantities could be selected for and monitored, it is not immediately clear how they relate back to network dynamics (i.e., patterns of neuronal firing) and the specific functional role of a network. As such, a network defined by these quantities does not necessarily provide a useful framework for understanding processing; neither does it yield straightforward strategies for network manipulation or control in plastic networks.

To create a consistent framework that helps explain network function and can elucidate strategies for modifying neural networks, it is desirable to develop an understanding of mechanisms for how interactions between single and groups of neurons give rise to network activity in a way that is tractable for analysis and relevant for the underlying function of the system. In light of these concerns, a framework that investigates the role of common topological features in plastic neural networks may provide substantial insight into how network dynamics arise from topological constraints, particularly in the context of network plasticity (Stone and Tesche, 2013; Whalen et al., 2013). There is evidence of common topological features at the microcircuit level (ranging from a handful of neurons to tens of neurons) (Milo et al., 2002; Douglas and Martin, 2004; Sporns and Kötter, 2004; Song et al., 2005), though few studies have investigated structure-function relationships in microcircuits that incorporate knowledge of the underlying dynamics of the circuit (Whalen et al., 2013).

Although STS was designed with classic two-neuron STDP in mind, the probabilities involved with randomly sampling multiple cortical sites implies at least a three-neuron network. We consider here how the simplest network elements—serial and parallel feedforward monosynaptic and disynaptic pathways—can be substrates for measurable network plasticity in the presence or absence of natural spiking correlations. The networks studied are the simplest that could conceivably give rise to STS effects. To systematically identify the contributions of various structural (topological) and dynamical (background activity) properties toward network manipulability, we constructed models of feedforward network topologies with spiking excitatory neurons exhibiting STDP and stimulated with STS. We then characterized the effects of network-wide perturbations upon the coupling between neurons in these small networks, revealing system parameter combinations that create STS effects.

## Methods

### Network model

**Figure 1:**
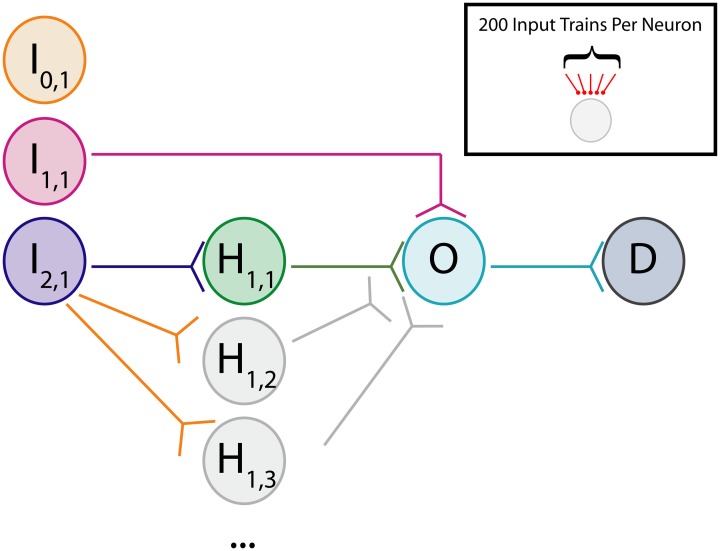
**Cartoon of network**. Schematic for augmented disynaptic feedforward pathway (aDFP) network structure. Shown are Input (I), Hidden (H), Output (O), and Downstream (D) neurons. The input neurons are either unconnected (I_0,1_; orange node), monosynaptically connected (I_1,1_; magenta node), or disynaptically connected (I_2,1_; blue node) to the output neuron (O; cyan). Every experiment has at least one disynaptic pathway with a hidden neuron (H_1,1_; green node). Additional identically connected Hidden neurons are only present in some experiments (H_1,2,…*N*_; gray nodes). Synaptic connections are all unidirectional.

To study neural dynamics in a tractable setting, a small feedforward network was constructed consisting of several identical excitatory neurons arranged in the pattern depicted in Figure 1. For these experiments, neurons labeled “I” are input neurons, neurons labeled “H” are hidden layer intermediate neurons, neurons labeled “O” are output neurons, and neurons labeled “D” are downstream from the output. Each neuron is designated first by its topological distance (i.e., number of synapses away) relative to the output neuron, and then by its copy number. For example, a neuron labeled I_2,1_ is two synapses distant from the single output neuron and it is the first neuron of its type. The network under study here comprises a disynaptic neuronal pathway from input to output (I_2,1_ → O), a separate monosynaptic pathway (I_1,1_ → O), and a disconnected “input” neuron for control experiments (I_0,1_) (Technically, the disconnected neuron is infinitely distant from the output neuron, but we designate it with 0 for notational convenience). Some experiments involve additional hidden layer neurons at multiple copy numbers. The terms “pre-synaptic” and “post-synaptic” in this case are always used in reference to output neuron O. We will refer to this general network architecture as an augmented disynaptic feedforward pathway (aDFP). Each of the neurons in this network simulation has identical dynamics:

$$C_{\text{memb}}\frac{dV_{\text{memb}}}{dt}=-\frac{\left(E_{\text{rest}}-V_{\text{memb}}\right)}{R_{\text{memb}}}+I_{\text{back}}+I_{\text{network}}+I_{\text{stim}}$$

These leaky-integrate-and-fire (LIF) dynamics capture subthreshold responses induced by background activity outside the network (*I*_back_), inside the network (*I*_network_), or from experimental stimulation (*I*_stim_). Each neuron “fires” an action potential when its voltage exceeds a threshold *V*_thr_ and has an absolute refractory period during which no action potential can be generated, given by τ_refrac_. Transmission of action potentials across synapses is accompanied by a synaptic delay *t*_delay,syn_ = 1 ms.

Synaptic input to a neuron (from afferent connections or artificial stimulation) is modeled using a simple alpha-wave characteristic of actual synaptic dynamics (Jack et al., 1975):

$$I=w\cdot g(t)=w\cdot te^{-\frac{t}{\tau_{\text{rise}}}},$$

where *w* is the weight of the synaptic input and *g*(*t*) is an alpha function. It can be shown that *g*(*t*) is a solution to a second-order linear differential equation. Thus, the evolution of *g*(*t*) can be reconstructed by numerically integrating the equivalent differential equation. As shown by Raman and Gutierrez-Osuna (2004), the corresponding second-order differential equation can be decomposed into the following first-order differential equations and integrated to recover *g(t*):

$$\begin{array}{l} \frac{dg}{dt}=-\frac{g}{\tau_{\text{rise}}}+z(t)\\ \frac{dz}{dt}=-\frac{z}{\tau_{\text{rise}}}+g_{\text{norm}}\delta (t) \end{array},$$

where *g*_norm_ is chosen to normalize the post-synaptic potential appropriately and δ(*t*) is the Dirac delta function, which evaluates to 1 when the pre-synaptic neuron fires and remains 0 otherwise. To provide baseline spontaneous activity throughout the network, every neuron in this network is driven by 200 separate background neurons. Each background neuron is modeled as a Poisson process with a rate of λ_back_. In this study, the manipulability of networks was considered in the presence of either purely uncorrelated inputs or inputs with a modest amount of correlation. Correlated background activity was constructed by introducing correlated spiking activity between any two neurons within a given time step using the correlation factor *c*_back_ (Gtig et al., 2003). This process results in background activity having a Pearson product moment correlation approximately equivalent to *c*_back_ within a time step but a correlation of approximately 0 between distinct time steps. Unless otherwise indicated, uncorrelated background activity was given a *c*_back_ value of 0, and correlated background activity was given a *c*_back_ value of 10^−3.5^.

These dynamics were simulated using a first-order forward Euler-based method in MATLAB with a time step of Δ*t* = 1 ms. All parameter values used in the simulation are given in Table 1.

**Table 1:** **Simulation parameters for LIF neural network model**.

**Parameter description (name)**	**Parameter value**
**LIF neuron parameters**	
Membrane capacitance (*C*_memb_)	200 pF
Membrane resistance (*R*_memb_)	100 GΩ
Resting voltage (*E*_rest_)	−70 mV
Threshold voltage (*V*_thr_)	−55 mV
Absolute refractory period (τ_refrac_)	2 ms
Synaptic delay (*t*_delay,syn_)	1 ms
**Synaptic waveform properties**	
Synaptic conductance waveform rise time (τ_rise_)	2 ms
Synaptic conductance normalization term (*g*_norm_)	0.000001
Excitatory synapse reversal potential (*E*_ex_)	0 mV
**Plasticity (STDP) parameters**	
Potentiation amplitude fit constant (*A*_*p*_)	0.001
Potentiation decay time constant (τ_*p*_)	20 ms
Depression amplitude fit constant (*A*_*d*_)	0.003
Depression decay time constant (τ_*d*_)	20 ms
Maximum synaptic conductance (*g*_max_)	6000 pS
**Input parameters**	
Input firing rate (*f*_input_)	0.8 spikes/s
Number of inputs processes per neuron (*n*_trains_)	200
**Spike-triggered stimulation (STS) parameters**	
Correlation delay	20 ms
Correlation probability	100% (50,000 pS)
**Simulation parameters**	
Simulation time step (Δ*t*_step_)	1 ms

### Synaptic plasticity rule

**Figure 2:**
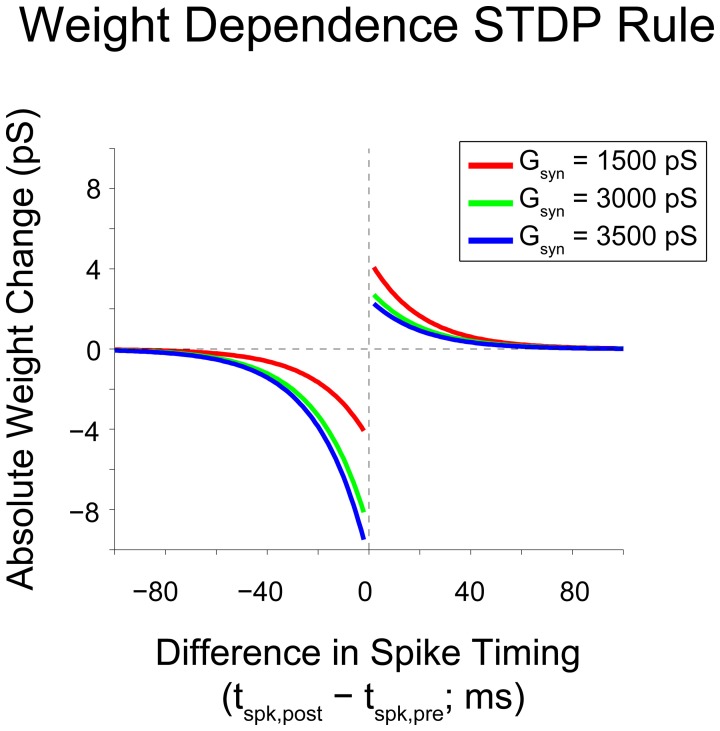
**Gütig weight rule visualization**. Visualization of a weight-dependent STDP rule for various synaptic weights. This variant of the STDP rule consists of two separate exponential curves with identical time constants (τ_*p/d*_ = 20 ms), and is scaled appropriately based upon the sign of the difference in spike timing between pre- and post-synaptic neurons (positive vs. negative). The weight dependence of the rule is implemented through a scaling of the time-dependent weight change by a linear or affine function of the current synaptic weight. As synaptic weight grows, the maximum weight change due to potentiation decreases and the maximum weight change due to depression increases. Based upon the weight dependence of the rule, a given synaptic weight stays bounded between 0 and *g*_max_ = 6000 pS.

Synaptic plasticity between real neurons takes many forms, and the rules that govern these plastic changes vary with neuron type and brain area. A well-studied type of synaptic plasticity exploited to induce functional changes in brain networks (see Introduction) is defined by the precise timing of action potentials in pre- and post-synaptic neurons influencing the magnitude and direction of change of the synaptic strength. This “fire together, wire together” class of rules is referred to as spike-timing-dependent plasticity or STDP (Markram et al., 1997; Bi and Poo, 1998; Song and Abbott, 2001; Froemke and Dan, 2002; Turrigiano and Nelson, 2004). The model neural network analyzed here incorporates a form of STDP with a stabilizing weight-dependent component to prevent synaptic strengths from collapsing or increasing without bound (Rubin et al., 2001; Gtig et al., 2003). This weight-dependent STDP rule is given by

$$\Delta w_{ij}=\{\begin{array}{c} A_{p}\left(g_{\text{max}}-w_{ij}\right)\cdot e^{-\frac{\left|\Delta t\right|}{t_{p}}},\Delta t>0\\ A_{d}w_{ij}\cdot e^{-\frac{\left|\Delta t\right|}{t_{d}}},\Delta t<0 \end{array},$$

where *w*_*ij*_ is the weight of the synapse from neuron *i* to neuron *j* and Δ*t* is the difference between spike times of neuron *j* and neuron *i* (i.e., Δ*t* = *t*_*j*_ − *t*_*i*_). The implementation of this weight rule in this network uses the latest spike generated by each neuron to update synaptic weights, a so-called “latest-neighbor” (Zhu et al., 2006) or “symmetric interpretation” rule (Morrison et al., 2008). A weight rule of this form was chosen because of its inclusion of weight-dependence, which has been observed experimentally (Bi and Poo, 1998), and because it yields stable network dynamics for networks of varying sizes (Rubin et al., 2001; Gtig et al., 2003). This stability is assured because of the inclusion of a soft upper bound on the synaptic conductance, *g*_max_. This soft bound prevents the synaptic weight from increasing uncontrollably through scaling the weight change of potentiation by the difference between the soft bound and the current synaptic weight. This scenario reflects the biophysical reality that synaptic conductances have a maximum value because transmembrane current cannot reach arbitrarily high values. To accurately reflect the assumption that plasticity-induced changes occur at the post-synaptic neuron, the STDP rule is defined such that weight updates occur after synaptic transmission of the pre-synaptic signal (i.e., it takes into account synaptic delay when considering the relevant pre-synaptic spike time for updating). Figure 2 shows the relation between spike timing difference and weight change for a variety of initial synaptic weights.

The shape of the function used to update the synaptic weight can take many forms and is observed to be dependent both on the type of neuron and the brain region in which the neuron resides (Abbott and Nelson, 2000). The shape of the weight rule used throughout these studies matches one that has been observed between neocortical excitatory cells (Froemke and Dan, 2002).

### Modeling of network perturbation by artificial stimulation

In traditional experimental paradigms testing STDP *in vitro*, current injections into the pre- and post-synaptic neurons are delivered at a fixed time difference and fixed repetition rate to induce changes in synaptic strength (Markram et al., 1997; Bi and Poo, 1998; Froemke and Dan, 2002). Attempts to exploit STDP *in vivo* using a more naturalistic paradigm by triggering stimulation at one site from recorded activity at another site have successfully altered function within primate motor cortex (Jackson et al., 2006). While inspired by the STDP rules at single synapses, the functional effect observed by Jackson et al. is clearly not confined to individual synapses but must be a manifestation of multiple synapses throughout the network. Using this phenomenon as a prototype for network-scale perturbation, our model implements a STS paradigm whereby the “stimulation” (output) neuron was triggered to fire action potentials *t*_delay_ = 20 ms later than the “recording” (input) neuron. STDP-associated synaptic changes in neocortex fall off exponentially with a time constant of ~20 ms (Bi and Poo, 1998; Froemke and Dan, 2002). To induce a reasonable change in synaptic strength, we selected a delay of *t*_delay_ = 20 ms for our model stimulation experiments. This delay has been reported to induce LTP for monosynaptic connections (Bi and Poo, 1998) and to increase similarity in neuronal responses between neurons isolated in separate locations (Jackson et al., 2006).

Artificial stimulation using injected current was modeled to a first approximation by considering the resulting depolarization to be identical to the activation of a synaptic conductance. The strength of this conductance increase, *w*_stim,max_ = 50,000 pS, causes the “stimulation” neuron to reach threshold and fire an action potential with 100% probability (i.e., so that every “recording” neuron spike results in a “stimulation” neuron spike). Thus, because the signal transmission for this protocol is deterministic once conditioned on “recording” cell firing, the perturbation caused by this stimulation is stronger than any of the other subthreshold probabilistic inputs that the “stimulation” neuron receives.

### Simulation protocols

Before performing experiments on an aDFP network, weights for the background processes were constructed with a 1-neuron simulation receiving projections from 200 background processes firing at 0.8 spikes/s. Once the evolution of these “background weights” stabilized (defined by at least 5000 s of no discernible change in the mean weights of all synapses), these derived weights were then used to generate a single realization of an aDFP network with background processes firing at 0.8 spikes/s. Following stabilization of synaptic weights in the network (“network weights”), this “base” network was run for 5000 additional seconds, followed by a 10,000 s experimental intervention and a 10,000 s post-interventional period. During all segments of the simulation, from generation of the aDFP network through the post-interventional period, background and network synaptic weights all remained plastic according to the full STDP rule as defined above.

Two types of manipulations were considered in this study. In one intervention, the firing rate of output neuron O was increased by increasing the background firing rates of projections onto this neuron from 0.8 spikes/s to 0.95 spikes/s. In remaining experiments, STS was performed by recording the spiking of a single input neuron (e.g., neuron I_0,1_) and triggering stimulation in neuron O following every spike from the input neuron during the entire 10,000-s interventional period. Weights and firing rates of individual synapses and neurons were sampled at 400 and 20 s, respectively, for visualization and figure generation.

### Metrics of functional change

**Figure 3:**
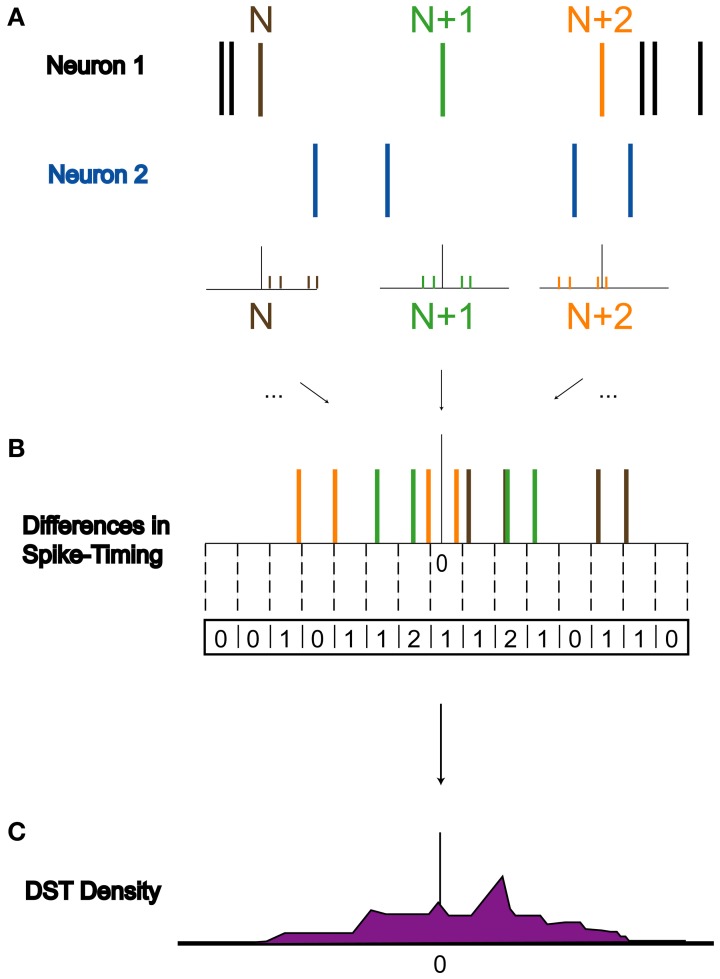
**Derivation of DST distribution**. Derivation of difference in spike timing (DST) distributions from spiking data. **(A)** Within a 10-s interval, DSTs are calculated for each pair of spikes in both spike trains. As shown, the time of the spike in Neuron 1's spike train labeled N is compared to the times of spikes in Neuron 2's spike train to generate a set of DSTs. **(B)** All of these DSTs within the analysis interval are then combined together and binned at 1 ms resolution. **(C)** Binned DSTs are normalized by the total number of DSTs to yield a DST probability density function.

To assess how perturbations to the network affect its dynamics, we quantified differences in spike timing (DSTs) due to changes in driving activity, as illustrated in Figure 3. Since STDP relies upon the relative timing between two neurons, it is reasonable to infer that differences in activity giving rise to changes in synaptic weights can be observed by changes in the distribution of DSTs measured between a pair of neurons. For a given experiment, multiple realizations of a network were created and used to rebuild the time-dependent distribution of DSTs. Data were then blocked into bins of 10 s for the calculation of DSTs and distributions were created with spike-time difference resolutions of 1 ms. To clearly elucidate the presence of structured spike timing relationships, all DST distributions were plotted as log probability vs. DST, except in Figure 11.

## Results

To better understand the effects of artificial electrical stimulation on perturbing cortical networks, we constructed a microcircuit model with a feedforward topology (shown in Figure 1; see Methods). This network, referred to as an aDFP network, was stimulated using a variety of protocols. This network is composed of an isolated neuron, a single monosynaptic connection, and a variable number of disynaptic connections from neurons in the input layer to the neuron in the output layer.

**Figure 4:**
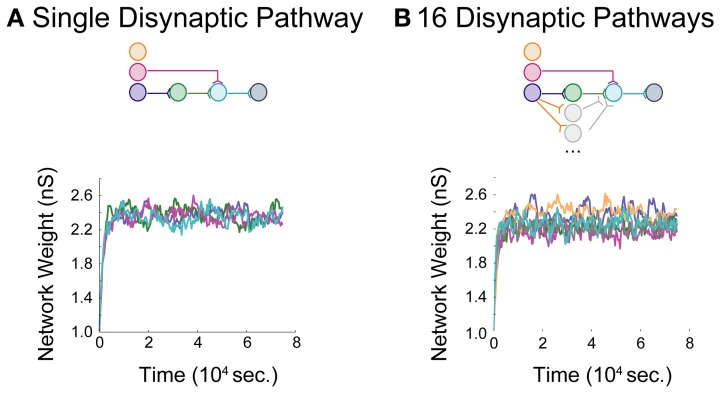
**Baseline model data for an aDFP network**. Baseline model behavior in the absence of correlated background activity. Color schemes for identifying neurons and synapses are the same as in Figure 1. Network insets reflect network topologies simulated in each panel. **(A)** An aDFP base network with one disynaptic pathway achieves steady-state network weight behavior by ~15,000 s. **(B)** An aDFP base network with 16 parallel disynaptic pathways achieves similar steady-state network weight behavior by ~15,000 s. A single representative parallel disynaptic pathway in this network is shown with a pair of orange and gray traces.

Networks with differing background correlations and topologies were simulated until steady-state dynamics were achieved. Prior to evaluating any given topology, a one-neuron network was initially constructed to derive a steady-state weight distribution among 200 background inputs onto the neuron, each spiking at 0.8 spikes/s. The resulting distribution of “background weights” was used to initialize weights for the background processes onto each of the neurons in each full network, and the full network was then simulated for 75,000 s to obtain stable steady-state dynamics in a single pathway configuration (Figure 4A) and a multipath configuration (Figure 4B). In each aDFP network without background correlation, the mean background weights settled to steady-state by 15,000 s of simulation time, exhibiting some random fluctuation brought about by the STDP rule during continued spiking activity. The weights of the synapses in the single-pathway network settled to slightly higher values than those in the multipath network. Before subsequent manipulations were performed, any 75,000 s “base” network was simulated for an additional 5000 s to allow for comparison both before and after manipulation.

### Effects of changes in output neuron firing

**Figure 5:**
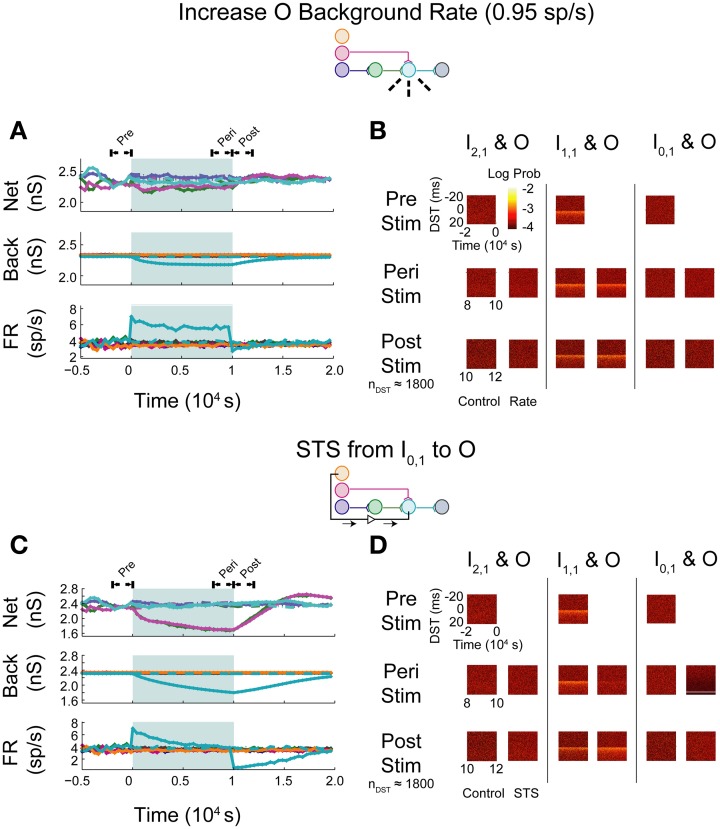
**Step increase in background FR + control STS of unconnected neuron pair in an aDFP network with uncorrelated background activity**. Stimulation of output neuron in the absence of background correlation causes depression of pre-synaptic weights. Color schemes for identifying neurons and synapses are the same as in Figure 1. Network insets reflect network topology tested and the experiment performed to generate the data in each pair of panels. **(A)** Network responses to a step increase in firing rate (to 0.95 spikes/s) of background processes onto neuron O, indicated by dashed lines on the network inset. Times of increased rates are indicated by the filled backgrounds. Time-varying synaptic weights (“Net” subpanel shows network weights; “Back” subpanel shows weights of background processes converging onto each network neuron) and network firing rates (shown in “FR” subpanel) are shown for control and intervention experiments (dashed and solid lines, respectively). Control and experimental simulations start from the same pre-intervention network and run for a 10,000 s peri-intervention period and a 10,000 s post-intervention period; the control network does not experience the same intervention (i.e., step increase in background firing rates) but runs the same amount of time so that a comparison can be made despite underlying variability due to random background activity. Each trace represents the average over *n* = 10 trials during the peri- and post-stimulus periods. Segments of simulation used for analysis in **(B)** are indicated by black arrows with terminating end-points above top panel. **(B)** Difference in spike timing (DST) probability distributions are measured between each of the input neurons and neuron O during various periods of the protocol. Each pair of columns gives the DST distributions for a particular neuron pair during control (left column in pair) and intervention (right column in pair). For the pre- and peri-stimulus periods, spiking data from *n* = 10 trials were combined to generate time-varying distributions; for the post-stimulus period, a number of trials were used to yield a constant number of *n* ≈ 1800 DSTs so that a comparison can be made between conditions where spiking rates for the output neuron vary. **(C)** Network responses in the uncorrelated network during STS from unconnected neuron I_0,1_ to the output neuron O. Recording and stimulation neurons are indicated on inset. Plot conventions match those used in **(A)**. Segments of simulation used for analysis in **(D)** are indicated by black arrows with terminating end-points above top panel. **(D)** DST distributions in uncorrelated network during STS protocol driving neuron O with neuron I_0,1_. Plot conventions match those used in **(B)**.

In the first set of experiments, we considered the behavior of an aDFP network containing a single disynaptic pathway driven only by uncorrelated background activity. We first evaluated the changes induced by small perturbations in background activity brought about by increasing the firing rates of some of the background processes. Simulations were performed in which the firing rate of background processes onto neuron O were increased from 0.8 to 0.95 spikes/s for 10,000 s (peri-intervention period) and then the background firing rates were reset back to 0.8 spikes/s for another 10,000 s (post-intervention period). For comparison, a set of control simulations were performed in which the same network used in the rate-increase experiments was simulated for the same amount of time (20,000 s total for both the peri- and post-stimulation periods). In the control network, the average network synaptic weights (“Net”; Figure 5A, top panel), background synaptic weights (“Back”; middle panel), and network firing rates (“FR”; bottom panel) fluctuated within a stable range throughout all periods (see Supplementary Figure S1A for control data plotted separately). In response to an increase in firing rate of the background processes for output neuron O, the weights of both synapses converging onto that neuron (from I_1,1_ and H_1,1_) transiently decreased, and the mean background weight persistently decreased (Figure 5A, top and middle panels). Moreover, the firing rate of neuron O increased briefly before settling to an intermediate value of ~5 spikes/s (Figure 5A, bottom panel).

As one method of assessing functional connectivity, the time-varying distributions of differences in spike-timing (DSTs) were constructed from 2000 s out of the pre-, peri-, and post-intervention periods of the protocol. DSTs in short time windows (5 s window length) were combined from multiple trials (10 for pre- and peri-stimulus periods and a variable number of trials to ensure a consistent DST count across conditions during the post-stimulus period). As shown in Figure 5B, an increase in firing rate alone did not obviously affect the spike-timing relationships between neurons. The DST distribution of the monosynaptic neuron pair showed high density at short positive latency, reflective of the strong interaction enabled by a single monosynaptic connection. Interestingly, no structure was visible in the DST distribution from the disynaptic pair. This result follows from low synaptic strength of the incoming network synapse from I_2,1_ to H_1,1_ and the relatively high number of uncorrelated inputs that are also being integrated at neuron H_1,1_.

The manipulability of interactions between neurons in different network layers was then investigated by using an artificial stimulation protocol to perturb network function. This stimulation was conducted by recording action potentials from one recording or source neuron in the input layer and, following each spike, delivering a depolarizing stimulus to a designated target neuron (for all cases, the output layer neuron) with a fixed delay (*t*_delay_ = +20 ms). This procedure is referred to as STS. As a control, STS was performed between an unconnected input neuron (I_0,1_) and the output neuron O. As shown in Figure 5C, application of STS coincided with marked depression in both network and background connections to the output neuron (top and middle panels). The firing rate of the output neuron settled lower compared to that observed during an increase in the background firing rate, to a value near the baseline rate of the neurons in the network. At stimulation offset, the network dynamics normalized to their pre-intervention activities; the network weights, however, overshot as they evolved toward their initial values (top panel). In addition, the spike timing relationship between the disynaptic pair was not obviously affected, while the relationship between the monosynaptic pair was diminished during STS (Figure 5D). For the unconnected pair, much of the DST density was centered at +20 ms, and much of the spike timing activity at positive DSTs disappeared during STS. During the post-stimulus period, little structure was observed in either the unconnected or disynaptic pair, but some structure was observed in the monosynaptic pair, showing recovery of the monosynaptic interaction within 2000 s.

As expected, simply increasing the firing rate of the output neuron did not result in systematic changes in spike timing relationships among any neuron pairs in the network. The STDP rule alone does enable changes in synaptic weights and overall firing rates, but these changes do not translate into new spike timing patterns across the network.

### Effects of artificially correlating across serial synapses

**Figure 6:**
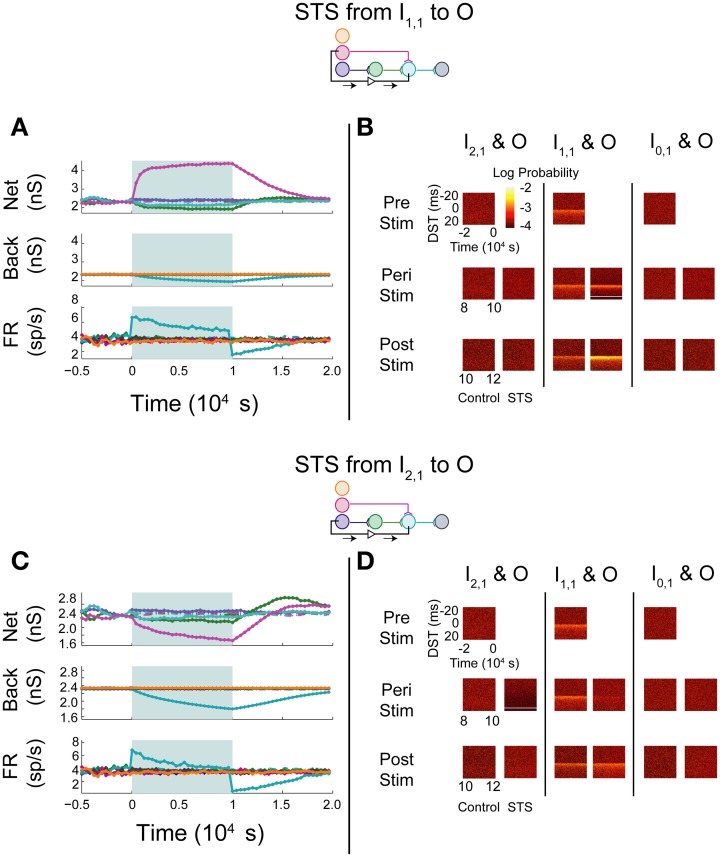
**STS in an aDFP network with uncorrelated background activity**. Monosynaptic and disynaptic STS in a feedforward network with uncorrelated background activity. Plot conventions are the identical to those in Figure 5. **(A)** Network response to STS from the monosynaptically connected neuron I_1,1_ to the output neuron O. A substantial increase in the synaptic weight flanked by the STS protocol was observed. **(B)** DST distributions during each phase of the STS protocol. An increase in probability of short-lag DSTs was observed. **(C)** Network response to STS from the disynaptically connected neuron I_2,1_ to the output neuron O. Synaptic weights terminating on the output neuron declined considerably. **(D)** DST probability distributions before, during, and after monosynaptic STS, as well as under control conditions where no stimulation occurred. A transient increase in short-lag DSTs was observed between the disynaptically connected neurons peri-stimulation, but this change disappeared post-stimulation.

STS was next performed between an input neuron connected to the output layer through one or more synapses and the output neuron. Stimulation of a monosynaptic pathway by recording from I_1,1_ and stimulating O increased the weight of the one synapse bridged by the manipulation, whereas the pre-synaptic connection from the hidden layer neuron H_1,1_ to O and the post-synaptic connection from O to D both decreased in weight (Figure 6A). The alterations in network weights changed with a time constant similar to or smaller than that observed in the unconnected case; the decrease in firing rate observed during stimulation, however, showed a longer time-constant. These changes persisted for a short amount of time after stimulation was removed, but returned to pre-stimulus conditions after approximately 5000 s. STS preserved the structure in the timing between the monosynaptically connected neurons during stimulation, and increased the density of DSTs at short positive lags (Figure 6B, middle column). The timing relationships between the other input-output neuron pairs were not visibly affected (Figure 6B, left and right columns).

Manipulation of neurons in a disynaptic pathway was modeled by recording from I_2,1_ and stimulating O. The network and background synaptic weights projecting onto and originating from the output neuron all decreased (Figure 6C), mirroring the effects observed while performing STS between the unconnected pair of neurons. Moreover, apart from the peri-stimulus artifact induced in the DST distribution between the disynaptic neuron pair, the timing relationship between each pair of neurons recapitulated the results in the STS protocol between the unconnected neurons. Persistence of effects following stimulation in both STS experiments was also short, and the network returned to baseline dynamics after approximately 7500 s (Figure 6D).

In this feedforward network with uncorrelated background activity, STS administered between unconnected input and output neurons yielded synaptic effects similar to those seen following a rate increase in the output neuron by scaling up background activity into that neuron. Both experiments were characterized by weakening of synapses onto the output neuron as its rate increased, consistent with the weight-dependent STDP rule. In the case of increasing background firing rate, however, the perturbation from initial dynamics was mitigated directly by slightly decreasing all of the background weights projecting onto the output neuron, lessening the influence of the perturbing dynamics on the output neuron. When STS was performed on a pair of unconnected neurons, modulating synaptic weights anywhere in the network did not alter the influence of the delivered stimulus upon the neuron. Thus, changes in activity and synaptic weights were much more pronounced in this STS experiment than for a simple rate increase, both inside and outside of the modeled network.

In general, STS in this network appeared to increase the coincidence of neuronal firing only when delivered between neurons that were monosynaptically connected, but appeared to decrease coincidence of neuronal firing when applied over neurons that were more distant from each other, even as little as two synapses away. STS applied between two monosynaptically connected neurons resulted in a strengthening of the synapse between them and a corresponding increase in the probability of spike-time pairs between them at small positive DSTs. This was an expected result because the STS protocol resembles the spike-pairing protocols used to elicit STDP in classic electrophysiology experiments. When the same STS protocol was applied to disynaptically connected neurons, however, the network response to this perturbation was much more similar to the network response when spike triggering originated from the unconnected neuron I_0,1_, with weakened network synapses and neuronal firing relationships. Notably, the H_1,1_-O synapse decreased relatively less than the I_1,1_-O synapse, which is different from the behavior during STS triggered by an unconnected neuron.

One trend that was consistent when performing either multisynaptic or unconnected STS was the presence of an overshoot in network weights ~5000 s after stimulation offset. This overshoot can be explained by noting that the influence of intra-network dynamics (measured by network weights) is greater than that of dynamics attributed to background activity. At stimulation offset, weights are tracking the firing statistics of the network and consequently increase above their pre-intervention levels as background weights recover with a longer time constant. During disynaptic STS, the synaptic weight from H_1,1_ to O overshot before and to a greater degree than the synaptic weight from I_1,1_ to O.

Under the conditions tested, then, the STDP rule appears to apply relatively independently to individual synapses. Another way of saying this is that neurons in this model greater than one synapse apart are relatively unconnected and do not greatly affect each others' firing patterns by themselves, though STS can potentially influence the transient recovery of networks by depressing the overall influence of background activity and increasing the role of intra-network dynamics. A scenario in which synapses operate independently therefore appears unlikely to lead to stimulation-induced functional remapping (Jackson et al., 2006).

### Effects of global network correlations

*In vivo* networks are not composed of strictly feedforward circuits with relatively weak functional connectivity between all neurons, and STDP in the context of a more richly interconnected network might result in stronger network-level effects. In order to avoid the additional complexities of recurrent network structures in the current series of experiments, yet capture some of the rich dynamics of real cortical networks, we next performed the same series of stimulation experiments on the same network topologies except with correlated background activity.

An aDFP network with modest global correlation in the background activity of all network neurons (global background correlation coefficient *c*_back_ = 10^−3.5^) was simulated. The combined influence of 200 background inputs with this correlation coefficient developed moderate levels of correlation between in-network neuronal spike trains (correlation coefficient of ~0.01). Global correlation is defined such that any two background processes are more likely to spike synchronously on average in a given time bin than if they were purely independent processes. This could be realized, for instance, by neurons in a primary sensory area that receive divergent projections from the same class of inputs responding to an external stimulus/cue.

**Figure 7:**
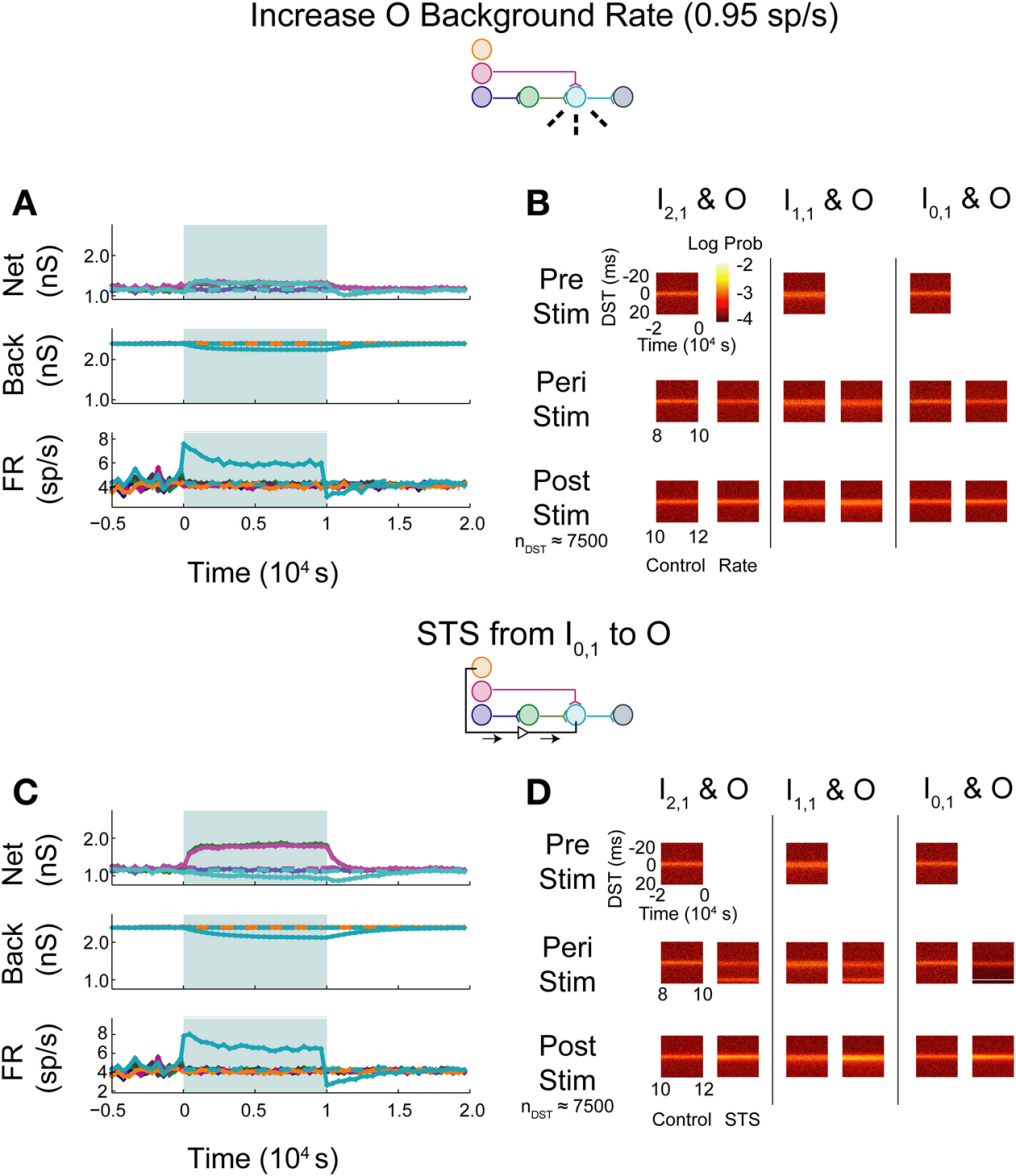
**Increase background FR + control STS in aDFP network with correlated (*c* = 10^−3.5^) background activity**. Stimulation of the output neuron in the presence of correlation shows increased potentiation of network weights. Plot conventions are the same as in Figure 5. **(A)** Network responses to STS in a network with background correlation to a step increase in the firing rates of background processes onto neuron O. **(B)** DST probability distributions before, during, and after monosynaptic STS, as well as under control conditions where no stimulation occurred. Frequency of short-lag DSTs increased for the monosynaptic and disynaptic input-output pairs, but little difference was observed for the unconnected neuron pair. **(C)** Network response of correlated network to STS from disconnected neuron I_0,1_ to output neuron O. All synaptic weights terminating on the output neuron increase peri-stimulation. **(D)** DST distributions in correlated network during each phase of the STS protocol driving the output neuron O with the unconnected input neuron I_0,1_. Short-lag DSTs increase peri-stimulation for all input-output neuron pairs.

Upon increasing the firing rate of background connections onto neuron O, minor increases in network weights were observed while background weights decreased systematically (Figure 7A; see Supplementary Figure S1B for control data without experimental data). In contrast to the uncorrelated case, timing relationships between neurons were not substantially affected by the increase in firing rate (Figure 7B), though timings in all three pairs, particularly in the monosynaptic pair, showed decreased DST density at short positive lags peri-intervention and increased DST density at short positive lags post-intervention.

During STS between the unconnected pair of neurons, both network synapses projecting onto the output neuron increased in weight while the outgoing synapse from the output and the background weights depressed (Figure 7C). In addition, the firing rate of the output neuron settled at an intermediate firing rate greater than its baseline, similar to the case in which the background firing rate was increased, but different from that observed with the similar experiment in the uncorrelated aDFP network. The timing relationships between input-output neurons were visibly changed during and after STS (Figure 7D). Increased DST densities at +20 ms during the peri-stimulus period appeared in all three of the DST distributions for the input-output neuron pairs, consistent with the presence of additional correlation in the circuit. Moreover, the density of short positive DSTs increased immediately post-stimulation for all three pairs (Figure 7D, bottom row).

**Figure 8:**
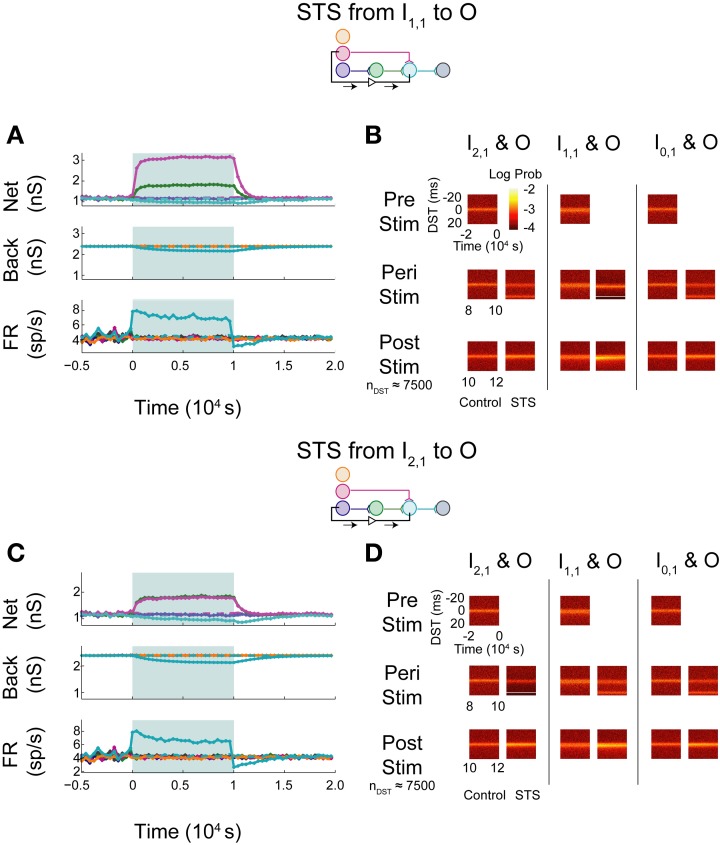
**STS in an aDFP network with correlated (*c*_back_ = 10^−3.5^) background activity**. STS is facilitated by correlation in background activity. Plot conventions are the same as in Figure 5. **(A)** Network response to STS from the monosynaptically connected neuron I_1,1_ to the output neuron O in the presence of correlated background activity (200 inputs with *c*_back_ = 10^−3.5^). All synapses terminating on the output neuron increased in weight. **(B)** DST distributions during each phase of the STS protocol. Probability of short-lag DSTs increased considerably for the monosynaptic pair. **(C)** Network response to STS from the disynaptically connected neuron I_2,1_ to the output neuron O in the presence of correlated background activity. **(D)** DST distributions in the correlated network during each phase of the STS protocol. Disynaptic STS is able to increase the frequency of short-lag DSTs for all input-output neuron pairs.

The simulated aDFP network with global correlation showed that stimulation of a monosynaptic pathway with STS leads to increases in both the targeted weight's connection (I_1,1_ to O), as well as other incoming weights onto the output neuron (H_1,1_ to O), as shown in Figure 8A. The weight of the outgoing connection from O to D decreased slightly. The firing rate of the output neuron also stabilized at an intermediate rate above its baseline, similar to the observations noted when STS was performed with the unconnected neuron pair. During monosynaptic STS, similar densities at +20 ms appeared during stimulation and greater short, positive DST densities were maintained for at least 2000 s post-stimulation when compared to the corresponding control condition (Figure 8B). Direct monosynaptic STS induced equal or greater changes in the density of DSTs for the monosynaptically connected neuron pair.

Increases in synaptic weights due to global correlation were also reflected under disynaptic stimulation conditions (Figure 8C), where STS induced increases not only in H_1,1_'s synapse with the output neuron, but also the ancillary monosynaptic connection from I_1,1_ (Figure 8C). The synaptic weight of the connection originating from the output neuron decreased by ~50–100 pS. The firing rate of the output neuron stabilized at an intermediate rate above its baseline rate. STS induced increases in short, positive DSTs between both monosynaptic and disynaptic neuron pairs, a change maintained for at least 2000 s (Figure 8D).

In general, the presence of zero-lag correlation created predictable changes in synaptic weight and spike timing during and after STS. Synapses projecting onto the output neuron potentiated because of the positive lag between the spiking of the pre-synaptic neurons (from the input and hidden layer) and the post-synaptic neuron that was induced by STS; conversely, the single synapse from O to D depressed as STS induced spiking at a negative lag between spikes generated by the O and D neurons (since now the pre-synaptic neuron O spikes 20 ms after the post-synaptic neuron D). Further, the original short-lag spike timing relationship (peak at +1 ms) observed between input and output neurons was disrupted during STS because a significant fraction of spike-time differences were now at +20 ms, effectively creating two smaller peaks in the distribution. After STS, however, increased intranetwork synaptic weights projecting onto the output neuron (generated during STS) now facilitated and strengthened the original spike timing relationship (increasing the peak at +1 ms).

In contrast with the previous experiments, STS between indirectly connected neurons with background correlation actually increased the frequency of DSTs within the network, both within and outside of the pathway connecting the neurons. This finding reflects a direct change in the spike-time relationship between the two neurons. When the network was driven by correlated background activity, the STS protocol was able to strengthen multiple pathways within the network simultaneously. Most notably compared to the uncorrelated case, disynaptic STS was able to strengthen connections converging onto the output neuron. STS was also able to create changes in the short-lag timing relationships between disynaptically connected neurons that persisted post-stimulation. Moreover, correlation between the neurons in the disynaptic pathway and the input neuron I_1,1_ allowed for simultaneous strengthening of the monosynaptic pathway converging onto the output neuron.

With shared correlation, STDP acts on similar timing properties seen within each pathway and was able to strengthen separate pathways simultaneously. The synapse originating from the output neuron, however, decreased in strength. Depression in the O to D synapse follows from the fact that, since neuron D fires at zero lag sufficiently often with respect to other network neurons, STS causes neuron O to fire 20 ms later than neuron D, causing depression due to the directionality of the plasticity rule. This property can be harnessed by STS to perturb the network and meaningfully change the functional relationships between neurons within and between pathways, but it is still constrained if the weight rule, like STDP, depends upon the order of spike timing.

### Effects of parallel feedforward projections

Based upon the performance of STS in the correlated aDFP network, we believed that a network topology with increased transmission probability of spikes between the input and output layers could recapitulate the results observed when the network received structured, zero-lag correlated background activity. We constructed another aDFP network to investigate this hypothesis by adding 15 parallel disynaptic pathways to increase topology-induced correlation between the input neuron I_2,1_ and the output neuron O, then excited this network with uncorrelated background activity. To prevent the confound of an increased rate at the output neuron due to additional synaptic pathways, a fraction of the background connections projecting onto O was removed (64 connections) to fix the firing rate of O at approximately 4 spikes/s, which is comparable to its spiking rate when only a single disynaptic path exists between I_2,1_ and O.

**Figure 9:**
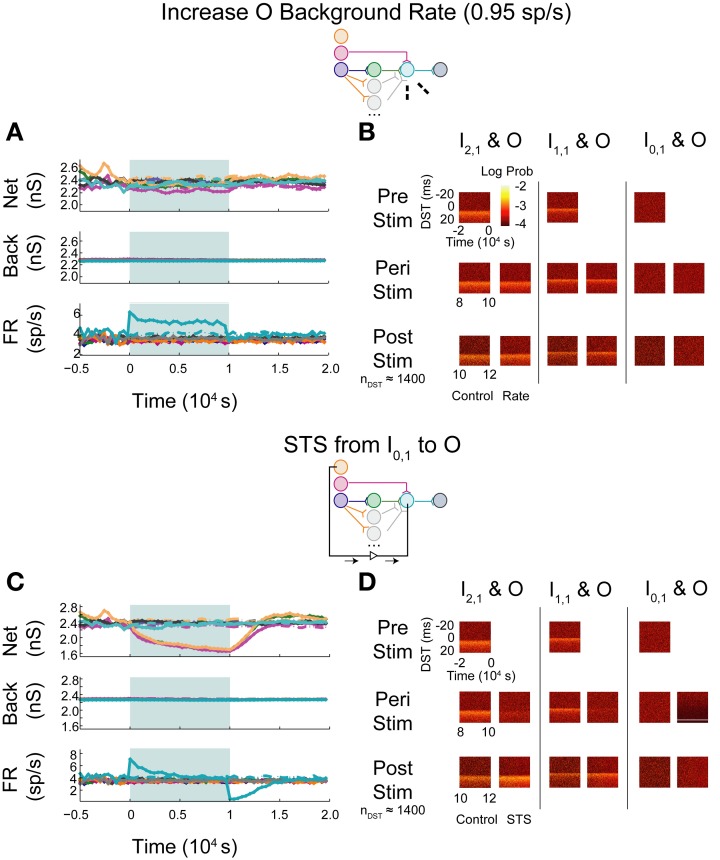
**Increase background FR + control STS in a multipath DFP network without correlated background activity**. Changes in network organization by rate increases and STS from the unconnected input neuron are mediated by multiple disynaptic feedforward pathways. Plot conventions are the same as in Figure 5. **(A)** Response of a multipath network between the disynaptically connected neuron I_2,1_ and the output neuron to a step increase in background firing rate of the background processes onto output neuron O. To compensate for the increased native rate in O due to increased intranetwork connections, 64 background connections were eliminated. **(B)** DST distributions for the multipath during each phase of the rate-increase protocol. **(C)** Network response to STS from unconnected neuron I_0,1_ to output neuron O in the multipath network. **(D)** DST distributions for the multipath network during the STS protocol.

When increasing the firing rate of the output neuron, little to no depression in background and network weights were observed (Figure 9A; see Supplementary Figure S1C for control data without experimental data). The baseline pre-stimulus activity showed a large density of positive DSTs between the disynaptically connected neuron pair at short lags, indicative of a structured timing relationship in the disynaptic circuit (Figure 9B, top left). Increased output neuron firing rate did not appear to have substantially changed the spike timing relationship between any pair of input and output neurons peri- or post-intervention. Alternatively, STS between the unconnected neuron pair showed similar trends in weights and firing rates (Figure 9C) and spike timing relationships (Figure 9D) as those observed in the single pathway network without background correlation. Moreover, STS with unconnected neuron pairs destroyed the structured timing relationship within the disynaptic pathway.

**Figure 10:**
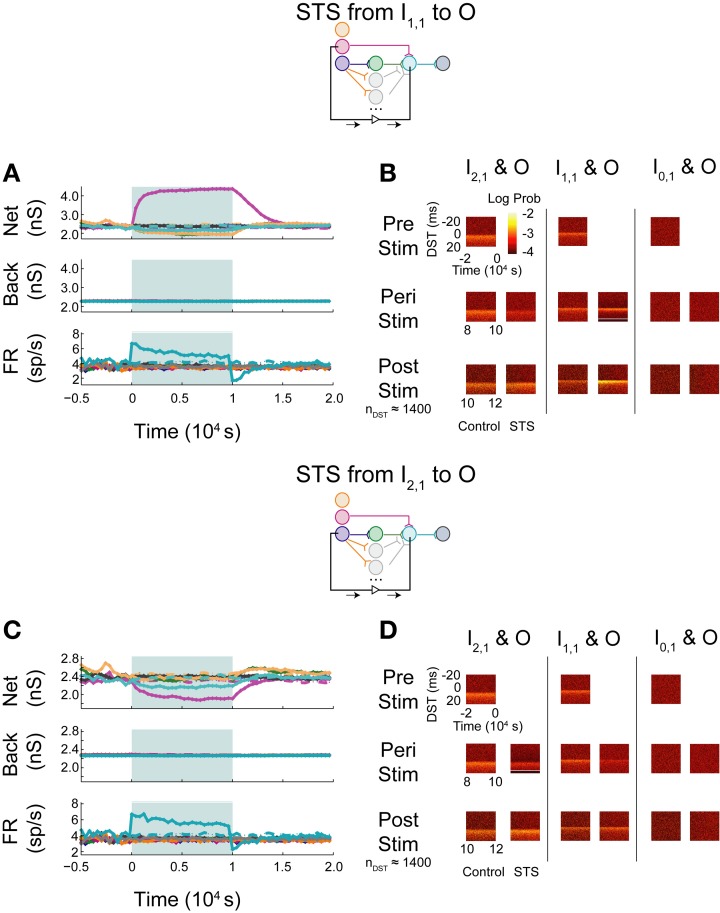
**STS in aDFP networks with varying hidden layer size**. Topologically induced correlation modulates STS in a multipath aDFP circuit. Plot conventions are the same as in Figure 5. **(A)** Network response to monosynaptic STS in the presence of 16 disynaptic feedforward pathways connecting I_2,1_ to O. The network response was similar to monosynaptic STS in the single disynaptic pathway network, but the synaptic weights converging onto the output neuron from hidden neurons in the disynaptic pathway decreased much less relative to the single pathway network. **(B)** DST distributions in multipath network during each phase of STS from the monosynaptically connected neuron I_1,1_, to the output neuron O. The frequency of short-lag DSTs diminished between the disynaptically connected neuron pair I_2,1_ and O. **(C)** Network response to STS from the disynaptically connected neuron I_2,1_ to the output neuron O. The qualitative response is the same as in the single disynaptic pathway network, but the synaptic weights converging onto the disynaptic neuron from hidden neurons in the disynaptic pathways decrease less than single pathway network. **(D)** DST distributions for the multipath network during each phase of the STS protocol. DST changes for the disynaptically connected neuron pair persist for ~2000 s post-stimulation.

When monosynaptic STS was performed on this network, the targeted monosynaptic connection increased in weight, as seen in the single pathway network, and the other intra-network synapses onto or originating from neuron O depressed (Figure 10A). Background connections decreased in weight, though much less than in the single pathway case. The decrease in firing rate observed at the output neuron is similar to that observed in the single pathway condition. During STS, the short DST timing was diminished between the monosynaptic pair but was still evident (Figure 10B, left columns), while, after STS little change from the baseline control condition was observed. Alternatively, in response to disynaptic STS, the synapse from the intermediate hidden layer neuron H_1,1_ remained high during STS (~2300–2400 pS in comparison to ~2200 pS in the uncorrelated case; Figure 10C). The synapse did, however, rebound and overshoot its steady-state value post-stimulation as it did in the single pathway case. Moreover, the hidden-layer synapse peaked faster after stimulation offset compared to its counterpart from I_1,1_ to O. The change in firing rate of the output neuron mirrored the change observed in the monosynaptic multipath case in Figure 10A. The timing relationship between the disynaptically connected neuron pair, however, did change due to stimulation. Post-stimulation, positive DST density at small positive lags increased by nearly a factor of two and broadened in the DST distribution of the disynaptic pair (Figure 10D, left columns). The DST distribution of the monosynaptically connected pair appeared to be minimally affected (Figure 10D; middle columns).

Monosynaptic dynamics in the multipath network were therefore similar to monosynaptic dynamics in the single pathway network, implying that monosynaptic behavior is relatively context-independent. For the disynaptically connected neuron I_2,1_ and the output neuron, however, a strong spike timing relationship at short, positive values was easily observed at steady state in the multipath network. In response to the disynaptic STS protocol, the DST density at short positive lag increased—a substantial difference compared with the single pathway network. Although the same qualitative dynamical behavior was observed in firing rates and synaptic weights, the absolute changes in these quantities during and after stimulation were much lower than in the single pathway network. This finding implies that the addition of multiple disynaptic pathways in the network induced a structured timing relationship between distantly connected neurons at baseline, as expected. Further, it also demonstrates that the dynamics induced by this topological feature under the given conditions is sufficient to allow systematic manipulation of spike timing relationships between distant neurons.

## Discussion

**Figure 11:**
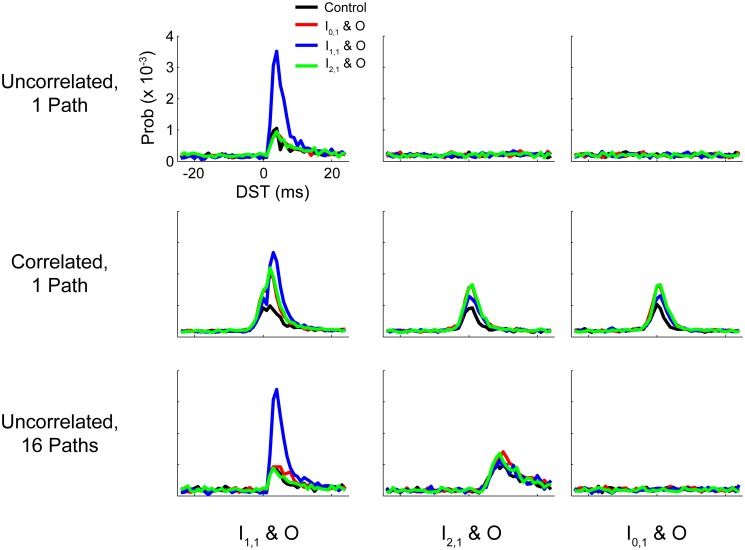
**Summary DST distributions for tested networks**. Summary of distributions of DSTs across all tested networks for the three input-output neuron pairs. Each distribution was generated by collapsing DSTs over the first 50 analysis intervals (250 s). Note that the scale of the ordinate axis is linear.

In this study we considered how the dynamics of simple plastic feedforward circuits were constrained by topological features and statistics of neuronal background activity. In particular, we studied how spike timing relationships and synaptic efficacies within the network could be modified during changes in network activity by perturbing the network with a simulated activity-dependent artificial stimulation protocol known as STS. STS has been shown to systematically alter neural network activity *in vivo* (Jackson et al., 2006; Rebesco et al., 2010; Guggenmos et al., 2013; Song et al., 2013) by delivering a stimulus related to the endogenous activity of the circuit, essentially acting as a short circuit or a high-efficacy synapse that takes advantage of structured spike timing relationships between targeted neurons. We demonstrated that baseline function and circuit responses to STS (particularly the strength and persistence of induced changes) were highly dependent on background statistics or topological features that induced correlated activity between input and output neuron pairs. A summary of the effects of these perturbations on circuit spike timing relationships is provided in Figure 11, and a quantification of these changes is provided in Supplementary Figure S2.

For all tested networks, monosynaptic connections showed strong STDP effects, changing both intranetwork weights and the spike timing relationships of the neurons involved in STS. This was expected for monosynaptic conditions because STS essentially yields fixed-lag spike pairings similar in principle to spike pairing used in classical STDP studies. Our ability to recapitulate computational and experimental results of STDP-inducing protocols with fixed-delay monosynaptic STS protocols in a network implies that the manipulability of monosynaptic behavior is relatively context-independent with respect to network conditions. Responses to disynaptic STS, however, varied with network input properties and network structure.

For the uncorrelated single-pathway network model, the outcomes of all non-monosynaptic STS paradigms were qualitatively similar to the outcome of the control condition where the inputs to the output neuron were step increased in rate. The changes observed in synaptic weights governed only by STDP under such conditions are therefore most likely due to chance collisions of spike events and not from timing-relevant spike transmission. These chance events are more likely during STS due to the increased rate brought about by the stimulation. In further support of this claim, disynaptic STS did not cause obvious changes in spike timing relationships despite causing weight changes.

When we added global correlation to the background neurons for the entire network, we saw the effects of STS across all paradigms, including STS from the unconnected network element to the output neuron. In this situation, the network-wide zero lag correlation may be thought of as a surrogate of common inputs to all network neurons. Thus, these observations show that the presence of common input provides a potential mechanism to take advantage of STS across multiple synapses.

The addition of multiple parallel pathways in the hidden layer of our model network caused a stronger initial relationship between I_2,1_ and O which could be sharpened (i.e., the preference for a particular spike time increased relative to others) through disynaptic STS. As shown in Figure 11 (last row, middle column), the post-STS effect for disynaptic STS increased the frequency of short-lag DSTs (+5 to +15 ms) for the disynaptic neuron pair compared to the control case in which no intervention was performed. When data were aggregated from the post-stimulus period over several analysis intervals, there was still surprising similarity in the distributions of DSTs for the disynaptically connected neuron pair between disynaptic and unconnected STS conditions. We believe that, although the effect is marginal, disynaptic STS was able to create a more selective change in DST frequency than unconnected STS; DST frequency reaches its maximum at +8 ms in the first case and falls off faster than the latter case where the peak DST frequency plateaus over the range of +9 to +11 ms. If we added more disynaptic pathways or included intrapathway correlation that was restricted only to the neurons in the parallel disynaptic pathways, we predict that we could discriminate the effects between disynaptic and unconnected STS more easily. In addition, compared to the single path network, changes in DSTs resulting from disynaptic STS in the multipath network were accompanied by much smaller changes in intranetwork weights, showing that the circuit more evenly distributed the overall induced changes among the available synapses from all the disynaptic pathways. Large network changes in such cases can therefore result from relatively small alterations in individual synaptic weights.

These observations suggest that polysynaptic feed-forward computation based on spike timing is substantially limited by the amount of shared correlation available to neurons in different layers. If such shared correlation exists, robust timing relationships can be sustained between polysynaptically connected neurons and, moreover, these relationships can be shaped by network perturbations (whether natural or experimenter induced). In light of the experimental observation that STS can successfully manipulate network function, it follows that manipulations of spike timing relationships across multiple synapses require coordinated network activity to realize this shared correlation, whether within pathways connecting two neurons or shared inputs belonging to both the recording and stimulation neurons. The success of STS under only some conditions tested implies that this kind of correlated activity exists in only some cortical microcircuits at any given time.

Several of the observations in these single and multipath networks depart from theoretical analyses of steady-state weight distributions performed in previous studies. A study using a similar weight rule and latest-neighbor spike-pairing scheme observed different qualitative behavior than this study (Zhu et al., 2006). In addition, unlike other computational studies that have investigated the effects of correlation in networks (Billings and Van Rossum, 2009; Gilson and Fukai, 2011), the presence of correlation within this network not only led to decreased baseline synaptic weights but also persistent times of induced changes after background activity reverted to pre-intervention levels. We attribute this mainly to the combination of complex dependencies of STDP on the type of weight rule considered, the spike-pairing scheme used to relate the order and number of spikes in spike trains to STDP changes, and the presence of synaptic delay (Burkitt et al., 2004; Zhu et al., 2006; Morrison et al., 2008; Babadi and Abbott, 2010). In particular, most previous analyses like those performed by Zhu et al. (2006) do not explicitly incorporate or discuss synaptic delay, though it is logical that delay would have a substantial impact on the neural processing modulated by a timing-based rule like STDP. In fact, a study by Babadi and Abbott (2010) found that introduction of synaptic delay into a single neuron model endowed with weight-independent STDP generated similar behavior (both in effects of correlation and increased firing rates on synaptic weights) to that observed here because the presence of synaptic delay on the order of 1 or 2 ms forced some of the monosynaptic interactions into the depressive side of the STDP window. The findings of Babadi and Abbott (2010) suggest that the discrepancies between this study and others could be attributed to the interaction between synaptic delay and STDP in our model. The details of the interactions between delay, the type of weight rule, and intranetwork correlation will likely require further investigation in experimental studies.

We observed one phenomenon that has not been previously reported in studies of STDP: transient overshoots or undershoots in network synaptic weights following STS. We believe that this phenomenon arises from the presence of multiple separate groups of synapses terminating on the output neuron (background neurons plus each individual network synapse) operating in distinct firing rate regimes at intervention offset. These groups capture the history of spiking activity in their synaptic weights due to the dependence of the number of STDP updates on pre- and post-synaptic firing rates. When the system is transiently changing, groups that fire quickly will update faster and can overshoot or undershoot as they compensate for slowly firing groups. Thus, the variety of timescales over which these changes occur between the different groups endows the system with memory (in the sense of dynamical systems). For instance, after performing STS between unconnected neurons (Figures 5A,B), the neurons projecting onto the output neuron from the monosynaptic and disynaptic pathways are firing more than the background processes (~ 4 vs. 0.8 Hz, respectively), but all of these synapses have comparable synaptic weights. On average, the increased activity of network neurons results in more STDP updates for network synapses than for background synapses. When network synapses reach their baseline values, background synaptic weights are still depressed compared to background weight baseline values; thus, network synapses increase in weight to compensate for decreased contributions of background synapses to output neuron activity and consequently overshoot. Network weights finally return to baseline after background processes recover and approach steady-state synaptic strengths.

When observing the effects of perturbation, we assumed that the primary indicator of network function consisted of spike timing relationships between network neurons. The primary analysis used to evaluate these relationships was the distribution of DST between pairs of neurons in the input and output layers. For neurons connected monosynaptically, this difference in spike times is directly related to the STDP calculation used to determine subsequent changes in synaptic weight. In response to variable background activity, DSTs are altered by STDP in response to these weight fluctuations and thus reflect the time-varying dynamics of the monosynaptic interaction. Even for non-monosynaptically connected neurons, this statistic has a direct physiological and functional relationship to temporal coding that the surrounding network may be utilizing. Several synapses away from peripheral sensory organs, it is still possible to reference the spike timing of a neuron relative to stimulus onset or to population generated signals such as local field potentials (Sukov and Barth, 2001; Vanrullen et al., 2005). This analysis metric captures and preserves physiologically and computationally relevant functional relationships between units within a neural network, though the question of how these temporal relationships relate back to the structural and functional properties of the network will most likely need to be elucidated in more complex networks.

Several assumptions in this set of experiments were used to perform a systematic yet tractable analysis of how perturbation may affect spiking timing relationships and synaptic efficacies in microcircuits. The most significant reduction was the use of extremely small circuits (on the order of tens of neurons). A valid concern for this study is whether the observations of small network dynamics are relevant to larger networks. For instance, with the enormous number of different network wiring configurations, it may seem unintuitive how studying so few neurons would contribute to understanding arbitrary structure-function relationships in neural circuits. Experimental findings, however, suggest that such an approach is logical. The presence of distinct motifs at the microcircuit level implies some level of non-random connectivity that is relevant for network function (Milo et al., 2002; Sporns and Kötter, 2004; Song et al., 2005). It is reasonable to believe that studying the dynamics of small motifs in isolation would inform their possible roles when composed together in large networks, allowing experimenters to disambiguate properties of network function caused by specific motifs and providing guidance for both theoretical and experimental investigations. In point of fact, most electrophysiological studies of plasticity investigate networks of two neurons (e.g., paired whole-cell recordings) and would not be practical for exploring networks of even the small sizes presented here, but their investigation yields fundamental principles for the function of these building blocks that can be useful for understanding larger networks.

Moreover, directly manipulating small circuits provides a greater understanding of the role of synaptic plasticity across multisynaptic topologies. In many computational analyses of plasticity in network function, the network considered either consists of a single neuron supplied by many synthetic background trains, or networks of hundreds or thousands of neurons randomly or totally wired together and driven by spontaneous background activity (Song and Abbott, 2001; Morrison et al., 2007; Billings and Van Rossum, 2009; Song et al., 2013). In a small group of neurons, non-trivial causal relationships between topology or background activity and overall network dynamics can be reliably inferred. Rather than just considering whether an intervention could make a change in an underlying network, we investigated what kind of changes could be induced in a small network and devised methods to understand how these changes are mechanistically related to features in that network (both in connectivity and induced spiking activity). By making this reduction, we performed investigations that could not easily be made in an experimental setting and made predictive hypotheses that inform experimental perturbations or manipulations of real neural networks. This work, nevertheless, warrants further consideration from future experimental studies, not only for validation of these observations in small biological neural networks but also for further exploration of the degree to which small-network principles can be extended to larger networks.

Another restriction we made in this study was restricting our scope of experiments to exclude topologies with recurrent connectivity. While many of the motifs of interest are likely to consist of recurrent connections, in order to attribute changes in network function to recurrent connectivity, it is first necessary to investigate which changes are inherent to the presence of feed-forward connectivity. One disadvantage in the study of small feed-forward networks is that these networks can lack highly structured activity (endowed by large-scale network structure) that serve as a meaningful reference for observing changes due to perturbation. To address this concern, we supplied our network with activity that was correlated within a time step, but not with other time steps. It has been thought that such a method of inducing synchronous activity requires balanced excitation and inhibition to complement its effects within larger networks. In a previous study, for example, the degree of excitation induced by zero-lag correlated background activity driving a network of ~10^5^ neurons modulated the network's steady-state between two regimes: either the entire network entered into a pathological regime if correlation was strong enough or selective neurons that were spontaneous correlated actually weakened as other intranetwork weights weakened in response to the induction of activity unrelated to the rest of the network (Morrison et al., 2007). These results suggest that proper tuning is necessary to provide suitable steady-state operating conditions in neural networks, particularly for a network operating with STDP since the dynamics at the synapse are controlled by many opposing potentiating and depressing updates. Thus, neural networks likely depend on the native correlations in background and network activity induced by the selective convergence of neural activity. While this concern is valid, we believe that the method used here to induce correlation is a reasonable first-pass approximation for understanding small network motif responses to structured background activity, and we expect to investigate how precise spike-time tuning can be achieved through topology when moving to more extensive network models.

One other observation from real neural networks that was not included in this study was the presence of inhibitory neurons. Inhibition is thought not only to control the amount of overall network activity by preventing overexcitation of excitatory neurons, but also to modulate the coincidence of neural firing and induce synchrony at the microcircuit and brain area levels (Buzsaki et al., 1992; Beierlein et al., 2000; Pouille and Scanziani, 2001). In light of these observations, it is possible that the coincidence needed to transmit information between neurons that are not monosynaptically connected could also be realized by having inhibitory pathways in the circuit that act on intermediary or output neurons to synchronize activity among layers. On the other hand, inhibition that disrupts the synchrony of a polysynaptic circuit could potentially decrease polysynaptic signal transmission and restrict network dynamics. Regardless, while inhibition is likely to be very important for generating complex neural dynamics, study of both network connectivity and plasticity of inhibitory neurons is lacking (Lamsa et al., 2010). A more rigorous analysis using inhibitory neurons is expected to be developed as greater understanding of inhibitory circuit dynamics and plasticity becomes available.

In addition, the synaptic rule that we considered here is a simple pair-based rule that does not consider higher-order effects due to multispike pairings or other factors such as frequency dependence. Compared to some triplet-based STDP rules, pair-based STDP tends to have weak dependencies on firing rate and deviates from experimental results at high firing rates (>20 Hz) (Sjostrom et al., 2001; Pfister and Gerstner, 2006). In networks with high firing rates, the predictions of network responses due to perturbation are likely to be skewed as these previous results suggest that the interactions between opposing LTP and LTD updates with respect to a single pre- or post-synaptic AP will not simply be linear, and activity-dependent perturbations such as STS that act as fixed delays to excite pre- and post-synaptic neurons will induce more complex changes due to the significance of these interactions. For our networks where the firing rate is low (<10 Hz), however, the deviation of modeling results from experimental results is approximately constant and is not likely to be qualitatively different (Pfister and Gerstner, 2006).

Another limitation in the rule we considered in this study is that it implicitly bypasses the experimental observation that LTP due to STDP requires post-synaptic depolarizaton (arising from concurrent synaptic activity for instance) (Sjostrom et al., 2001; Clopath et al., 2010). This observation is potentially problematic when considering the effects of network perturbations on synaptic efficacy because it indicates that the precise temporal activity of one synapse can affect others within a local region of the dendritic tree. In a network with correlated background activity, STDP's dependence on post-synaptic activation is likely to be irrelevant because widespread global correlation among so many processes alone can induce the activation necessary to support LTP. On the other hand, in a network with only topologically induced correlation that is not precisely tuned, this dependence may be a substantial factor when very few afferents are activated or present; as suggested by Sjostrom et al. (2001), this situation may apply when as many as 4–8 afferents are concurrently activated but not with any constancy. Thus, the effects observed in the uncorrelated multipath network, while seemingly substantial, may be subject to the same stipulations. It is also difficult to compare the qualitative characteristics of weight-dependent rules such as the STDP rule used in this study to those formulated in many of these more nuanced studies because these studies frequently ignore contributions due to the synaptic weight (Froemke and Dan, 2002; Pfister and Gerstner, 2006; Clopath et al., 2010). In light of the complex contributions of myriad factors (e.g., frequency of stimulation, spike-timing dependence, voltage dependence, multi-spike interactions), it is likely that a greater biophysical understanding of plasticity mechanisms is necessary to elucidate the precise effects of each of these factors on real neural networks [see Karmarkar and Buonomano (2002) and Rubin et al. (2005) for examples of relevant biophysical modeling experiments].

While observations in this study may be limited by these aforementioned assumptions, they potentially have utility in improving experimental investigations into manipulating neural networks (i.e., with STS paradigms) and other investigations seeking to elucidate network function by experimenter perturbation. Manipulation of small or large networks with artificial electrical stimulation may be constrained by network function as seen here. For instance, one criterion that may increase manipulability using STS is that the electrical stimulation should be delivered in the presence of some correlated network activity (either stimulus-induced or related to neural dynamics). This could be achieved by presenting a natural (i.e., non-electrical) stimulus to which two neurons are receptive and establishing a robust timing relationship in the spiking activity of the two neurons related to the stimulus, or even further enhanced by behavioral tasks. A previous study tested this hypothesis (Ahissar et al., 1992) and showed that the degree of plasticity in auditory cortex, measured in response to an *in vivo* protocol similar to that considered here, was dependent upon whether the protocol was performed in the presence of behavior that was related to the stimulus feature (in this case, an auditory discrimination task between pure two different tones or between tones and band-pass noise). Moreover, these results imply that a more sophisticated STS-like protocol could be constructed that would interact with both network dynamics as well as external activity to achieve some desired control output. Such a protocol however, could be hindered by substantial increases in native networks rates (Figure S3), where the level of background activity, if not correlated to intranetwork activity, can dilute the effects of the protocol. It is also conceivable that the degree of induced change will vary with the probability of the applied artificial electrical stimulation to elicit spiking at the target site. Preliminary data for networks similar to those considered here in which this STS protocol was performed (uncorrelated monosynaptic and disynaptic networks) suggest a lowered probability of eliciting spiking would decrease the maximal inducible change and increase the time constant of change (data not shown).

Alternatively, particular cortical dynamics could be reinforced by triggering within relevant dynamical states. For instance, neural processing related to resting-state functional activity could be selectively reinforced by controlled stimulation during periods of increased activity in areas thought to be influential in activity of that subnetwork (Raichle et al., 2001). Stimulation could also be triggered from detected activity in a large-scale recording modality such as EEG. For example, increases in gamma wave (30–100 Hz) power coinciding with the negative phase of delta wave (2–4 Hz) activity has been shown to be related to increased population spiking activity (Whittingstall and Logothetis, 2009).

A second criterion for increased circuit manipulability may be the presence of network connectivity that would be predicted to reinforce signal propagation between two sites. This could consist of, for instance, the presence of many parallel pathways between neuron pairs or, on large-scales, different brain areas. If a circuit is predominately feed-forward, it may be possible to boost the timing relationship between two neurons in a pathway by finding and performing STS on neurons that are intermediate in the pathway and highly interconnected.

Alternatively, methods using perturbation to infer the underlying connectivity of a network may be limited by similar constraints as those seen here. A recent study by Lepage et al. (2012) used mean-field approximations of cortical columnar processing to give a proof of concept for such a perturbation-based circuit mapping method. As opposed to neuroanatomical studies, such methods capture the functional connectivity of the network, which is inherently biased by the ongoing activity in a circuit. Such a network mapping technique is likely to capture functional interactions that will approximate the real connectivity of a network, but is conditioned upon the processing of the network during observation and may need to be refined based on the range of network activity in the circuit.

This study investigated how network function is constrained by exogenous activity and network connectivity in small feed-forward networks. Using this framework, a set of principles was elucidated that constrains the dynamics the network is capable of attaining, namely, that feed-forward polysynaptic computation necessitates intrapathway and intranetwork correlations to transform network input, whether the source is from network input or topological structure. These principles are potentially useful for understanding how compositions of these small microcircuits actually operate *in vivo*, and they also present a preliminary framework for understanding and controlling neural networks more broadly. In light of the large sizes of native biological neural networks, future investigation into network dynamics will need to extend such frameworks to understand how different network components function together to realize transformations on their inputs. Further research into principles of control at a network level and the design of experimentally driven stimulation/perturbation protocols will likely form a foundation for understanding general network dynamics.

### Conflict of interest statement

The authors declare that the research was conducted in the absence of any commercial or financial relationships that could be construed as a potential conflict of interest.
