# Supplementary material for: Spike-timing computation properties of a feed-forward neural network model
Source: Front Comput Neurosci. 2014 Jan 28;8:5. doi: 10.3389/fncom.2014.00005 (PMC3904091; doi:10.3389/fncom.2014.00005)
Supplement: Supplementary file 1 [file Presentation1.PDF]

Supplementary Methods:

As a quantitative measure of the change induced by the various interventions in these experiments, we used a measure of the difference between pre- and post-intervention DSTs. We defined the intervention effect, IE, as a summed absolute difference between DSTs before and after intervention within the window of -25 to +25 ms:

$$IE = \sum_i \left| DST_{post,i}(0) - DST_{pre,i}(t_{end}) \right|$$

To generate DST_{pre} and DST_{post} respectively, we collapsed DSTs over the last 50 analysis intervals from the pre-stimulation period and first 50 analysis intervals from the post-stimulation.

For a general intervention, the number of possible induced changes is numerous, including from changes occurring at any single DST lag or any combinations of lags. Framing a DST distribution as a vector, this corresponds to changes in any direction of this 50-dimensional space (since the DST distribution over all lags is normalized to sum to unity, this makes it a subspace of one less dimension than the total number of components of the vector). We considered the "direction" defined by the pre-intervention DST to be the most relevant reference for understanding the changes induced by these interventions. We defined the effect direction, ED, as the normalized inner product between the pre-intervention DST and the effect difference:

$$ED = \frac{\left\langle DST_{post,i}(0) - DST_{pre,i}(t_{end}), DST_{pre}(t_{end}) \right\rangle}{\left\| DST_{post,i}(0) - DST_{pre,i}(t_{end}) \right\| \cdot \left\| DST_{pre}(t_{end}) \right\|}$$

The effect direction is a coefficient that ranges from -1 to 1, and measures that degree of similarity between the effect difference and DST_{pre} in a linear sense. We must, however, take into account the fact that DST_{pre} is inherently biased - the true “reference” or null distribution that corresponds to no spike timing relationships is the uniform random distribution. Mathematically, this refers to the fact that the center of the 50-dimensional subspace of DSTs is not at the origin (corresponding to the zero vector). Thus, prior to calculating the effect difference, we subtracted the quotient of total area of the DST_{pre} density function between -25 and 25 ms and the number of elements in DST_{pre} from each component of the DST to account for this consideration.

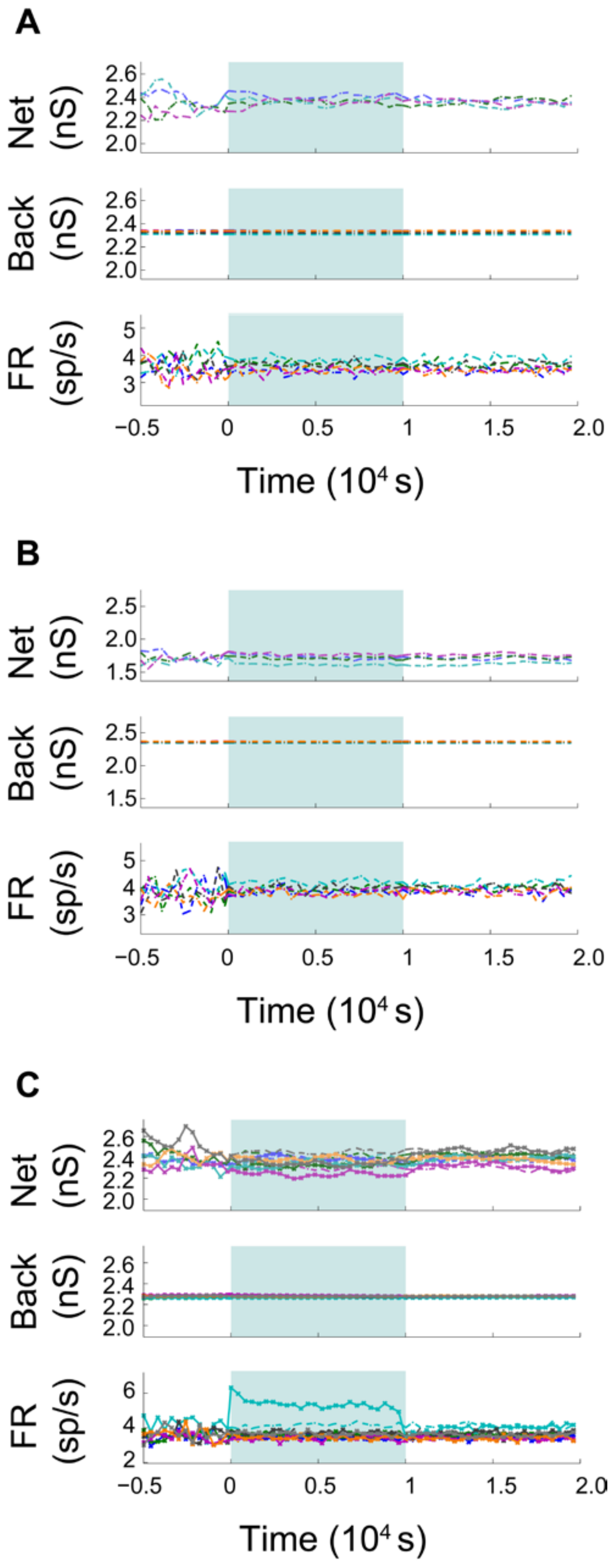

Figure S1: Control data for synaptic weights and network neuron firing rates for (**A**) the uncorrelated, single pathway network, (**B**) the correlated, single pathway network, and (**C**) the uncorrelated multipath network. All data have the same conventions as Figures 5-10 and are identical to the control data shown these figures in the main manuscript.

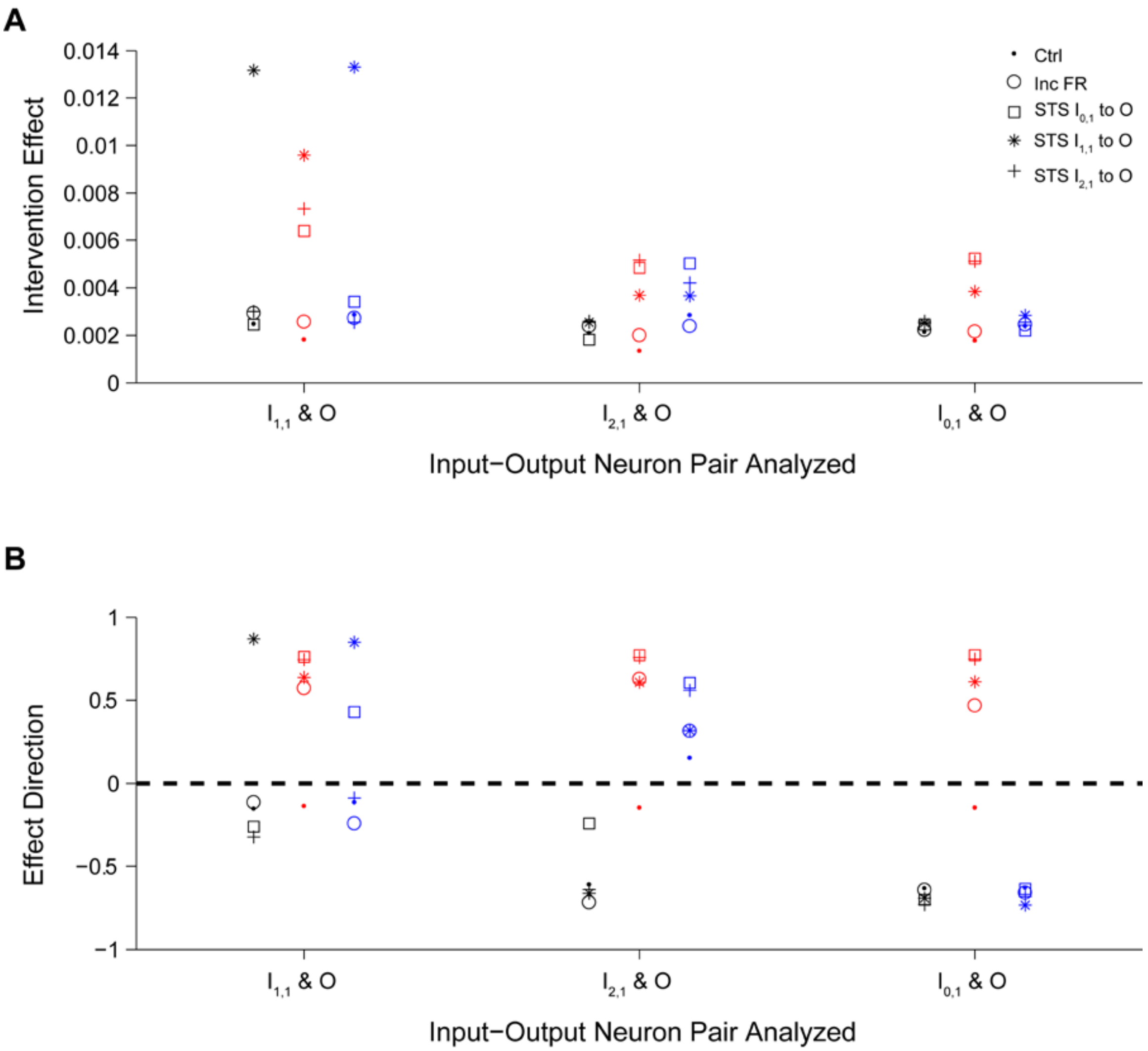

Figure S2: Summary statistics for changes induced by perturbations for all networks and interventions tested. (**A**) Intervention effect for all networks and interventions for each input-output pair. The intervention effect is greatest for the monosynaptically connected neuron pair due to monosynaptic STS. Intervention effects for unconnected and disynaptic STS interventions increased in the uncorrelated multipath network relative to the uncorrelated, single pathway network. Intervention effect for the unconnected neuron pair are indistinguishable for all interventions within each tested network, but induced a greater change in the globally correlated network. (**B**) Effect direction is a normalized measure of how the intervention effect preserved the structure observed in the pre-stimulation DST. A positive value indicates the intervention effect reinforced this structure, a negative value indicates that intervention reinforced the opposite structure, and zero value indicates that the induced difference is orthogonal to pre-intervention structure.

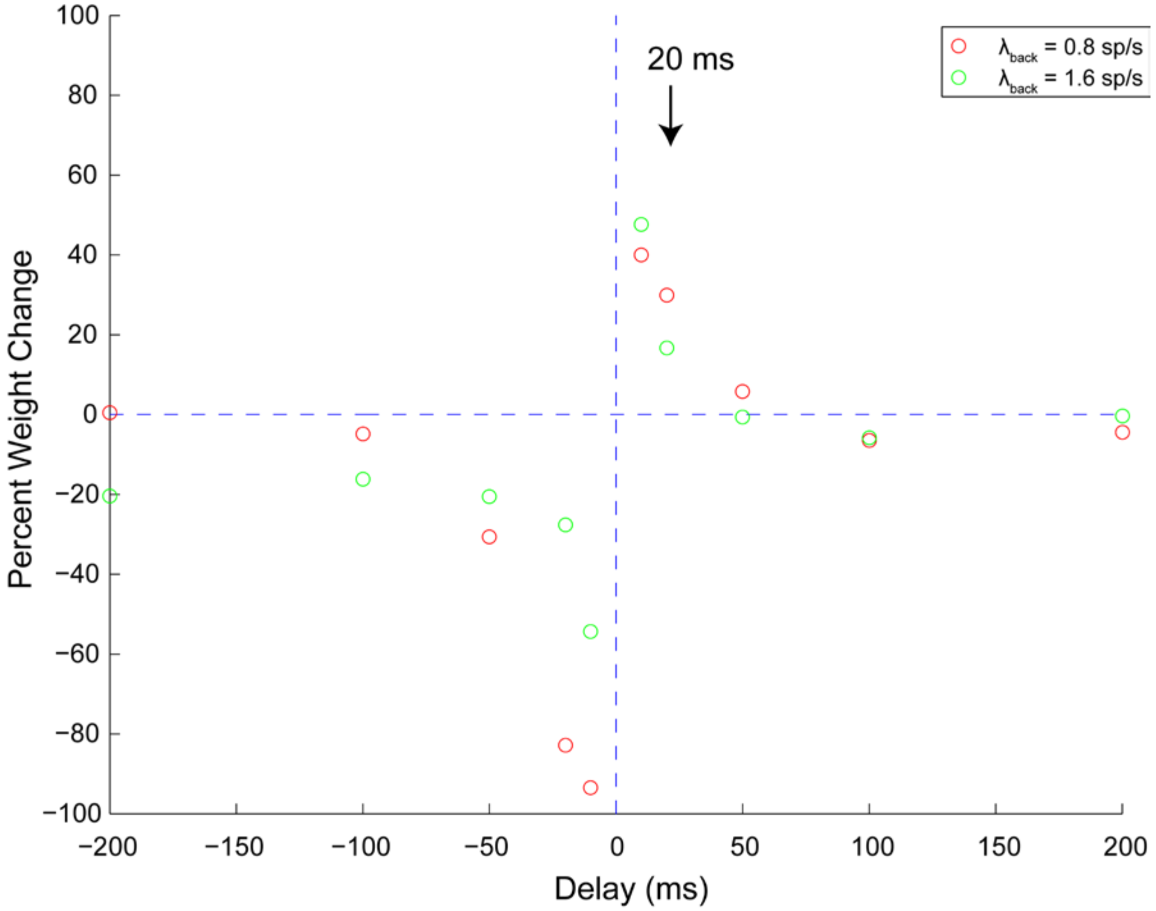

Figure S3: Effect of monosynaptic STS diminishes with increased background firing activity. A circuit of two neurons connected by single synapse with the same dynamics and synaptic learning rule as all other experiments was simulated with background firing λ_{back} = 0.8 or 1.6 spikes/s (red and green circles, respectively) at various stimulation time lags, and percent weight change was evaluated at the end of 2000 s of STS with the presynaptic neuron as the recording neuron and the post-synaptic neuron as the stimulation neuron. Each data point represents the outcomes of a single trial. The arrow denotes a stimulation time lag of 20ms, used in all other experiments in this study. In general, increased background firing rate tends to diminish the degree of synaptic weight change; at stimulation lag of 20ms, the percent change in synaptic change induced by STS dropped by half between the low and high firing rate conditions. The post-synaptic neuron was firing at ~ 3 spikes/s in the low firing rate condition and ~16.5 spikes/s in the high firing rate condition during the stimulation period.
